# Comparison of Transthoracic Echocardiography (TTE) and Transesophageal Echocardiography (TEE) Guidance for Transcatheter Aortic Valve Replacement (TAVR) Performed Under Conscious Sedation: A Retrospective Study

**DOI:** 10.7759/cureus.105335

**Published:** 2026-03-16

**Authors:** Ramlah Khan, Emad Ramadan, Mohamad Taha, Alexander Sbrocchi, Subhash Banerjee, Robert C Stoler, Saravanan Ramamoorthy

**Affiliations:** 1 Department of Internal Medicine, Baylor University Medical Center, Dallas, USA; 2 Department of Anesthesiology, Texas A&amp;M Naresh K. Vashisht College of Medicine, Bryan, USA; 3 Department of Cardiothoracic Surgery, Baylor Scott & White Heart and Vascular Hospital, Dallas, USA; 4 Department of Cardiology, Baylor Scott & White Heart and Vascular Hospital, Dallas, USA; 5 Department of Anesthesiology and Perioperative Medicine, U.S. Anesthesia Partners, Dallas, USA; 6 Department of Anesthesiology, Baylor University Medical Center, Dallas, USA

**Keywords:** aortic valve replacement, outcomes, transcatheter aortic valve replacement, transesophageal echocardiography, transthoracic echocardiography

## Abstract

Transcatheter aortic valve replacement (TAVR) is increasingly performed using long-acting sedation instead of general endotracheal anesthesia (GETA), offering benefits such as shorter procedure times, fewer complications, and quicker recovery. Intraoperative echocardiographic guidance is critical for procedural success, with transesophageal echocardiography (TEE) offering superior imaging quality compared to transthoracic echocardiography (TTE). However, TEE is less commonly used with long-acting sedation. This study assessed the feasibility and safety of TTE vs TEE in TAVRs under long-acting sedation. This retrospective observational study analyzed 60 TAVR cases performed at a quaternary care center, with 30 guided by TEE and 30 by TTE. All procedures were performed under long-acting sedation. Procedural metrics, imaging quality, and in-hospital outcomes were compared between groups. The two groups were similar in baseline characteristics. Procedure times did not significantly differ (TEE: 70.46 ± 36.59 minutes vs. TTE: 66.44 ± 25.51 minutes, p = 0.624). However, TEE provided significantly superior imaging quality (mean score 5.0 vs. 2.73 ± 0.83, p < 0.001). No significant increase in complications or need for escalation to GETA was observed in the TEE group. TEE can be safely and effectively used to guide TAVR under long-acting sedation, offering superior intraoperative imaging without prolonging procedure time or increasing risk. This approach supports the continued expansion of long-acting sedation-based TAVR protocols using TEE in appropriate patients.

## Introduction

Transcatheter aortic valve replacement (TAVR) has emerged as an intervention for patients with severe aortic stenosis who are at all levels of risk for conventional surgical valve replacement [1,2]. As TAVR continues to be applied to a broader spectrum of patients, precise intraoperative imaging remains critical to ensure procedural success, minimize complications, and optimize patient outcomes [1,2]. Initially, TAVRs were done under general endotracheal anesthesia (GETA), but there has been a shift from GETA to long-acting sedation due to faster procedure times, fewer procedural complications, and a more favorable post-procedural course [3,4].

Echocardiography has an increasingly important role in TAVR procedures, providing real-time imaging guidance during valve deployment and assessment of post-procedural valve function. With the increasing number and type of TAVR valves, intraoperative imaging becomes imperative in optimizing valve function and minimizing complications.

The two primary echocardiographic modalities used in TAVR are transesophageal echocardiography (TEE) and transthoracic echocardiography (TTE). TEE offers high-resolution, real-time imaging, but given its more invasive nature, it tends not to be used with long-acting sedation. TTE, performed externally, which makes it less invasive, offers lower quality and less timely imaging. With the increasing use of long-acting sedation in TAVR, the use of TEE, which was the original imaging modality to guide TAVR, has largely been replaced by TTE [3,4]. TEE is valuable for confirming valve placement depth, identifying paravalvular leaks, and assessing the extent of valve expansion [5,6].

This study aimed to compare TEE and TTE for guiding TAVR performed under long-acting sedation. We aimed to assess procedural feasibility, intraoperative image quality, procedural duration, fluoroscopy exposure, and in-hospital safety outcomes in a large quaternary system TAVR program.

## Materials and methods

Study population

This is a retrospective observational study analyzing data from 60 TAVR procedures performed at Baylor Scott & White Heart and Vascular Hospital in Dallas, Texas, over four months. Patients were analyzed in two groups based on the intraoperative imaging modality used: TTE guidance (n = 30) and TEE guidance (n = 30). Inclusion criteria for the TEE group were suboptimal preoperative imaging on TTE. Exclusion criteria for the TEE group were histories of esophageal surgery or known esophageal pathology.

Anesthesia protocol

TEE- and TTE-guided TAVR procedures were performed under total intravenous sedation. Anesthesia medications included dexmedetomidine 0.5-0.7 mcg/kg/hour and propofol 50-100 mcg/kg/minute. TEE-guided TAVR was performed with nasal continuous positive airway pressure (nCPAP) with 10 cm H₂O of inspiratory positive airway pressure, 5 cm H₂O of expiratory positive airway pressure, FiO₂ 100%, and oxygen flow rates of 10 L/minutes. To minimize inter-provider variability, all cases were managed using a standardized anesthesia protocol and intraoperative imaging protocol, following the standard TAVR echocardiography protocol with standard views, including color and Doppler imaging. The activated clotting time target was ≥250 seconds. Intraoperative imaging was continuous and interpreted collaboratively by a sonographer, the anesthesiologist, and the primary interventional cardiology team. Intraoperative monitoring included a left radial arterial line and a right internal jugular line with a single-lumen 6 French catheter and transvenous pacer wire. SEDLine was used to determine the depth of the anesthetic, which remained in the Patient State Index range of 25 to 50 during the procedure for both groups [7].

Device and implantation procedure

All patients underwent TAVR using either the Medtronic Evolut Pro valve (Medtronic, Minneapolis, MN) or the Edwards Sapien III valve (Edwards Lifesciences, Irvine, CA). Valve selection, including manufacturer and size, was determined by the cardiologist based on preoperative computed tomography (CT) assessment, including aortic valve and annular calcium scores.

Procedural planning was guided by pre-procedural imaging, including TTE and CT to evaluate annular dimensions, valve morphology, and vascular access. During the procedure, fluoroscopic imaging was used to ensure accurate catheter positioning and valve deployment, while echocardiography was used for intraoperative assessment of valve positioning and function, detection of paravalvular leak, and identification of potential complications.

Access for valve delivery was obtained via the transfemoral approach. Systemic heparinization was administered to maintain anticoagulation. Deployment was performed under fluoroscopic and echocardiographic guidance, with baseline hemodynamic measurements obtained pre- and post-deployment. Final positioning, stability, and absence of significant paravalvular leak were confirmed by TEE or TTE.

Access site closure was achieved using vascular closure devices, Perclose (Preclose technique) (Abbott Vascular, Santa Clara, CA) and Angio-Seal (Terumo Interventional Systems, Somerset, NJ). Protamine was administered to reverse anticoagulation, and all patients were monitored for hemodynamic stability and neurological integrity before transfer to the ICU or telemetry unit.

Data collection

Data were collected from electronic medical records, including age, gender, body mass index, prior cardiac procedures, demographic information, and outcomes. Procedural data included procedure time, fluoroscopy duration, and postoperative complications. The quality of the TTE and TEE studies was determined by experienced cardiac sonographers.

Statistical analysis

Continuous data were expressed as mean ± SD. Technical and procedural success rates were calculated as percentages of the total number of patients. For proportions, numbers and percentages were used. The TEE and TTE group differences were evaluated using the Student’s t-test for continuous variables. A p-value < 0.05 was considered significant.

## Results

Patient population

A total of 24 women and 36 men were included in this study, with an average age of 75.55 years, and cases performed between January 2022 and April 2022. Baseline characteristics of the 60 patients are summarized in Table 1. A total of 30 were guided by TEE, and 30 were guided by TTE. The clinical characteristics between the two groups were comparable. Four patients who underwent TEE had pacemakers, and one patient who underwent TTE had a pacemaker. No patients in either group required a pacemaker after the procedure.

**Table 1:** Baseline Characteristics of Patients Who Underwent TAVR Under TEE or TTE Guidance Continuous variables are presented as mean ± SD and compared using the independent samples t-test (df = 58). Categorical variables are presented as n (%) and compared using the chi-square test (df = 1). AF = atrial fibrillation; CHA2DS2-VA = congestive heart failure, hypertension, age ≥75 years, diabetes mellitus, prior stroke or transient ischemic attack or thromboembolism, vascular disease, age 65 to 74 years; df = degrees of freedom; eGFR = estimated glomerular filtration rate; LVEF = left ventricular ejection fraction; TAVR = transcatheter aortic valve replacement; TEE = transesophageal echocardiography; TTE = transthoracic echocardiography

	TEE (n = 30)	TTE (n = 30)	Test statistic	p-value
Age, years	76 ± 6	75 ± 10	0.47	0.45
Women, n (%)	9 (30)	14 (47)	1.76	0.11
LVEF, %	55 ± 11	56 ± 12	-0.34	0.83
Permanent AF	0	0	-	-
Creatinine, mg/dL	1.6 ± 1.5	1.3 ± 1.4	0.80	0.39
eGFR, mL/minute/1.73 m²	60 ± 26	65 ± 24	-0.77	0.40
CHA2DS2-VA	4.3 ± 1.3	4.1 ± 1.8	0.49	0.75

Procedural results

Procedural characteristics in the TEE and TTE groups are shown in Table 2. Procedure times were similar between the two groups, with TEE-guided procedures averaging 70.46 ± 36.59 minutes and TTE-guided procedures averaging 66.44 ± 25.51 minutes (p = 0.624). Image quality in the TEE group had an average score of 5 (excellent diagnostic quality) compared to 2.73 ± 0.83 in the TTE group (p < 0.001). 

**Table 2:** Procedural Characteristics and Complications and In-Hospital Outcome of Patients Who Underwent TAVR Under TEE or TTE Values are mean ± SD or %. Image quality scale: 1 = poor; 2 = suboptimal (challenging to interpret); 3 = acceptable (limited study); 4 = good; 5 = excellent (clear and diagnostic-quality images) Variables were compared using the independent samples t-test (df = 58). df = degrees of freedom; TAVR = transcatheter aortic valve replacement; TEE = transesophageal echocardiography; TTE = transthoracic echocardiography

	TEE (n = 30)	TTE (n = 30)	p-value
Procedure time, minutes	70 ± 37	66 ± 26	0.62
Fluoroscopy time, minutes	23 ± 8	18 ± 5	0.01
Image quality	5 ± 0	2.7 ± 0.8	0.01

Fluoroscopy and in-hospital outcomes

Fluoroscopy time was longer in the TEE group (23 ± 8 minutes vs. 18 ± 5 minutes, p = 0.01), reflecting higher-quality imaging that required more frequent valve repositioning and post-deployment balloon dilation, contributing to increased exposure. No major postoperative complications were observed other than minimal pharyngeal discomfort, which resolved within 24 hours. 

## Discussion

The primary finding of this study is that TEE can be used to guide TAVR safely and effectively in patients receiving long-acting sedation without the requirement of GETA. TEE-guided TAVR provides superior image quality, allowing precise valve deployment and minimizing potential intraoperative complications without a significant difference in procedure time [6]. While TTE is less invasive, there are limitations in real-time imaging, which can impact clinical outcomes. Imaging with TTE may provide limited visualization during the procedure, making it suboptimal for detecting perivalvular leak, aortic dissection, valvular depth, left atrial appendage thrombus, and other features contributing to long-term outcomes of TAVR [8].

TAVR is currently done under either GETA or long-acting sedation, with an increasing shift towards the use of long-acting sedation [1]. Outpatient TEEs are routinely performed under long-acting sedation with same-day discharge [9]. The theory behind this study was that TEE is done safely using long-acting sedation in the outpatient setting, so it should be safe to perform during long-acting sedation in patients undergoing TAVR. In our study, all TAVR cases were performed under long-acting sedation. Advantages of using sedation over general anesthesia during TAVR have been previously documented and include shortened procedure time due to avoidance of intubation and extubation, decreased respiratory complications, fewer vasopressors, and reduced ICU and hospital length of stay [4].

TEE can safely and effectively be used to guide TAVR in patients receiving long-acting sedation without GETA. TEE offers dynamic and precise intraoperative imaging, making it superior to TTE. Additionally, TEE-guided TAVRs noted in this study were done with nCPAP, which delivers a steady stream of oxygenated air, allowing airways to remain open, which has been shown to prevent intraoperative hypoxia [9]. The use of TEE should be considered to guide TAVR under long-acting sedation as a safe, effective, and clinically advantageous approach, offering superior imaging for precise valve placement while avoiding the risks associated with GETA.

Study limitations

This study's retrospective design and single-center setting limit generalizability. The small sample size (30 patients per group) reduces statistical power and introduces potential bias, including selection bias, as TEE was preferentially used in patients with suboptimal pre-procedural TTE imaging, as well as operator-dependent variability in imaging interpretation and intraoperative decision-making. The study evaluated in-hospital outcomes only and therefore cannot assess mid- or long-term valve performance or clinical outcomes. Additionally, anesthesia-specific outcomes such as cumulative sedation dose requirements and intraoperative hemodynamic parameters were not systematically compared between groups due to the retrospective design. Future prospective studies with larger cohorts and longitudinal follow-up could further evaluate these parameters.

## Conclusions

In this retrospective single-center study, TEE guidance during TAVR performed under long-acting sedation was feasible and associated with significantly superior intraoperative image quality compared with TTE, without prolonging procedural duration overall. Additionally, no significant differences in perioperative complications or pacing requirements were observed. These findings suggest that TEE may be safely integrated into TAVR workflows performed under long-acting sedation in selected patients, particularly when enhanced intraoperative imaging is desired, without significantly compromising procedural efficiency. Larger prospective studies are warranted to further evaluate the impact of TEE-guided TAVR under long-acting sedation on procedural outcomes, complication rates, and long-term clinical endpoints.
